# Translational methods in biostatistics: linear mixed effect regression models of alcohol consumption and HIV disease progression over time

**DOI:** 10.1186/1742-5573-4-8

**Published:** 2007-09-19

**Authors:** Mariel M Finucane, Jeffrey H Samet, Nicholas J Horton

**Affiliations:** 1 Department of Mathematics and Statistics, Smith College, Northampton, MA, 01063, USA; 2 Clinical Addiction Research and Education (CARE) Unit, Section of General Internal Medicine, Department of Medicine, Boston University School of Medicine and Boston Medical Center, Boston MA, 02118, USA; 3 Department of Social and Behavioral Sciences, Boston University School of Public Health, Boston, MA, 02118, USA

## Abstract

Longitudinal studies are helpful in understanding how subtle associations between factors of interest change over time. Our goal is to apply statistical methods which are appropriate for analyzing longitudinal data to a repeated measures epidemiological study as a tutorial in the appropriate use and interpretation of random effects models. To motivate their use, we study the association of alcohol consumption on markers of HIV disease progression in an observational cohort. To make valid inferences, the association among measurements correlated within a subject must be taken into account.

We describe a linear mixed effects regression framework that accounts for the clustering of longitudinal data and that can be fit using standard statistical software. We apply the linear mixed effects model to a previously published dataset of HIV infected individuals with a history of alcohol problems who are receiving HAART (n = 197). The researchers were interested in determining the effect of alcohol use on HIV disease progression over time. Fitting a linear mixed effects multiple regression model with a random intercept and random slope for each subject accounts for the association of observations within subjects and yields parameters interpretable as in ordinary multiple regression. A significant interaction between alcohol use and adherence to HAART is found: subjects who use alcohol and are not fully adherent to their HIV medications had higher log RNA (ribonucleic acid) viral load levels than fully adherent non-drinkers, fully adherent alcohol users, and non-drinkers who were not fully adherent.

Longitudinal studies are increasingly common in epidemiological research. Software routines that account for correlation between repeated measures using linear mixed effects methods are now generally available and straightforward to utilize. These models allow the relaxation of assumptions needed for approaches such as repeated measures ANOVA, and should be routinely incorporated into the analysis of cohort studies.

## Background

The National Institute on Alcohol Abuse and Alcoholism estimates that more than 13 million Americans suffer from alcohol dependence or abuse [1]. HIV infection has major health consequences, with estimates of 940,000 infected Americans [2]. These two health concerns are related, and alcohol problems have been reported to be more prevalent in HIV-infected patients. Among 665 patients who were establishing primary care for HIV infection, half were determined to have an alcohol problem based on the CAGE questionnaire or clinical assessment [3]. Before the advent of highly active antiretroviral therapy (HAART), however no association between alcohol use and HIV disease progression was found [4]. Samet et al. [5] hypothesized that in the age of HAART, alcohol use, because of its potential interaction with a variety of HIV clinical issues including medication adherence, might accelerate HIV disease progression. They found that, among a cohort of HIV-infected individuals with a history of alcohol problems (the HIV-Alcohol Longitudinal Cohort, or HIV-ALC), those individuals receiving HAART and consuming alcohol had significantly higher viral RNA (ribonucleic acid) levels at baseline.

In this paper, we provide a tutorial on linear mixed effect models to study repeated measures in this dataset including follow-up data collected on subjects in the HIV-ALC cohort [6]. Longitudinal cohort studies have the advantage of providing detailed information about how a given set of variables changes over time in an individual patient and of facilitating the study of the factors that influence this change. By collecting repeated measurements, we gain the ability to distinguish between the degree of variation across time for one person (within-individual change), and the variation among people (between-individual change). However, longitudinal studies present some statistical complexities, since the customary assumption that all observations are independent usually does not hold.

In addition to the usual assumptions of regression methods, models for a single outcome assume that all observations of a particular variable are independent of one another: knowing the value of one observation of a variable provides no information about the others, after controlling for known covariates. This assumption does not hold true in longitudinal studies, however, as multiple observations of a variable on a particular person are likely positively correlated (i.e. the errors may reflect a systematic trend within each individual).

One approach to this problem involves excluding all followup data from the analysis and using only the baseline data from the cohort – in this single-time-point subset of the original dataset, the assumption of independence of observations is plausible. However, this method utilizes only part of the available data, and is highly inefficient and inadvisable. Unless the correlation is quite high between baseline and follow-up data, such an inefficient approach will lead to less precise estimates, and does not allow for assessment of time-varying exposures and outcomes.

Another approach is to assume that repeated measurements on an individual are independent despite the fact that they are likely correlated. This may introduce bias into the estimates of variability of the models' parameters, and is not recommended (see [7] for a case study of the perils of this mis-modeling).

A more principled approach, which we will illustrate in this paper, involves modeling the within-individual relatedness (clustering) of measurements in order to make use of all the data and simultaneously obtain unbiased estimates of parameter variability. Although models that take this clustering into consideration are more complicated, they are also more powerful since they facilitate the study of change over time. These issues have received a great deal of attention in the statistical literature in recent years, and the books by Diggle and colleagues [8] and Fitzmaurice, Laird and Ware [9] provide excellent overviews of the field.

A classic method used to account for repeated measurements in linear models is repeated measures analysis of variance (RM-ANOVA). This model was developed for settings with discrete covariates, complete data, and common measurement occasions for all subjects [9]. This approach has some disadvantages in practice, however, since in many longitudinal studies observations may be unbalanced and/or incomplete and assumptions regarding equal covariance between all observations may not be tenable [10]. As we will illustrate, other approaches, such as the linear mixed effects model that we describe, are more attractive in this setting.

In this paper, we describe the linear mixed effects (LME) or random effects/random coefficients model of Laird & Ware [11], a versatile model that accounts for clustering. Other approaches to estimation in this setting are discussed by Fitzmaurice et al. [9]. The LME approach provides a flexible yet parsimonious way of modeling the association among repeated measurements. These within-subject associations are often of secondary interest in longitudinal studies and the parameters that describe them in the LME model are thus termed *nuisance parameters*. The *substantive parameters *are those that describe the relationship of primary interest between study variables. The LME approach estimates the nuisance parameters and substantive parameters simultaneously, yielding consistent estimates of the substantive parameters if the model for the covariance and the mean are appropriately specified.

Samet et al. [5] conducted a cross-sectional analysis of the baseline data from a cohort of HIV-infected individuals with a history of alcohol problems (HIV-ALC). In a multiple linear regression model that controlled for a number of potential confounding variables, they found that among subjects who were on HIV medications, those subjects who used any alcohol (moderate or at risk use) had significantly higher mean viral log RNA levels (p = .006) than subjects who reported no drinking (fully abstinent) during the previous 30 days; this association was attenuated (p = 0.04) when adherence to HIV medications was included as a predictor in the model. Further analysis of data from this cohort has been reported [6].

In this paper, we will fit LME models to conduct a secondary analysis, using longitudinal methods to further explore the issues they considered in their baseline analysis. The goal of our analysis will be to explicate linear mixed effects models in the context of understanding the association between alcohol consumption and the progression of HIV/AIDS. Using repeated measures data will allow us to take full advantage of all information available in this cohort study and to assess how the associations between alcohol use and HIV RNA levels changed over time. Use of the LME is preferable to other approaches such as classical RM-ANOVA, because it allows loosening of assumptions that may not be tenable. While these methods are particularly well-suited to the analysis we consider, they are also applicable to many other types of longitudinal epidemiology studies.

## Analysis

### Methods

We perform a secondary analysis of the HIV-Alcohol Longitudinal Cohort (HIV-ALC), a follow-up study of HIV-infected patients with past or current history of alcohol problems. The primary purpose of this longitudinal cohort was to examine HIV progression of these subjects, and prior results have been published previously [12-14]. Participants were recruited between July 1997 and July 2001. All participants resided in the Greater Boston area and were recruited through the following sources: Boston Medical Center (BMC) Diagnostic Evaluation Unit (56%), posted fliers (17%), BMC Primary Care Clinic (13%), respite facility for homeless persons (5%), methadone clinic (4%), subject referrals (4%), and Beth Israel Deaconess Medical Center (BIDMC) (2%). Persons recruited outside BMC or BIDMC were pre-screened by telephone, and potentially eligible individuals were invited to complete the screening process in person. The Institutional Review Boards (IRB) of BMC, BIDMC and Smith College approved this study.

Patients who were HIV-infected and had a history of alcohol problems were identified by explicit eligibility criteria: confirmed HIV infection and a history of alcohol problems. Patients not receiving care at BMC or BIDMC were asked to document their HIV diagnosis by providing either HIV testing documentation or their HIV prescription medications. Clinical assessment (in 10% of participants) or 2 or more positive responses to the CAGE questionnaire (in 90%) were used to identify participants with the criterion 'history of alcohol problems.' The CAGE questionnaire [15] is a short, validated questionnaire with good reliability in identifying problem drinkers. Diagnostic interviews for alcohol problems in a sub-sample of CAGE-positive subjects (n = 141) revealed a lifetime history of alcohol dependence (80%) or abuse (15%) [3]. Additional entry criteria included the following: evidence of unimpaired cognitive function as determined by a score of 21 or more on the Mini Mental State Examination [16]; no plans to leave the Boston area during the subsequent 2 years; and fluency in English or Spanish. For the Spanish interview instrument, standardized scales in Spanish were used when available; the remainder of the questionnaire was translated from English into Spanish, back-translated and revised.

There were 444 eligible subjects screened at these various sites, 350 (79%) provided informed consent and agreed to participate in the study. After providing informed consent, subjects were scheduled to be interviewed every 6 months for a maximum of 7 visits. Since no follow-up occurred after July 2001, subjects enrolled late in the study had only a baseline assessment and a small number of follow-up visits. Laboratory values of HIV RNA measured within 3 months of each visit were obtained from medical records whenever available. If not measured during routine clinical care, blood samples were drawn during the visit by nursing staff and tested for HIV RNA level. Participants were compensated US$20 or an equivalent gift certificate to a local grocery store. In this paper we analyze the subset of this cohort made up of all participants receiving highly active antiretroviral therapy (HAART) at baseline (n = 197).

### Outcome variable

The base 10 log of one plus the viral load of HIV RNA (log(RNA+1)) was used as a primary outcome of HIV disease progression in the HIV-ALC study. Measurement of HIV RNA was performed using branched-chain DNA techniques [17]. The lower threshold for detection at the time of the study was 50 copies/ml – values < 50 were analyzed as 0. We note that this approach is somewhat ad-hoc, and more principled approaches have been developed [18].

### Measures of alcohol consumption

In the HIV-ALC study, alcohol use in the 30 days before each interview was used as a measure of the usual pattern of use. To encourage accurate reporting of alcohol consumption, breath alcohol level was also measured before the interview [19]. Alcohol consumption was calculated using alcohol quantity and frequency questions as well as the Addiction Severity Index, an assessment instrument with well-documented reliability and validity in this population [20]. Alcohol use was initially classified as 'abstinent', 'moderate', or 'at-risk', based on the National Institute on Alcohol Abuse and Alcoholism (NIAAA) recommendations, which define at risk drinking as more than 14 drinks per week (or more than four in one day) for men, and more than seven drinks per week (or more than three in one day) for women [1]. Any alcohol consumption below these levels was considered moderate use in this study. Following the approach of Samet et al [5], a dichotomous indicator of any consumption (yes/no) was used in this analysis. Alcohol use was measured at each study visit and thus varied over time.

### Measures of adherence to HIV medication

Adherence to HAART was self-reported using the AIDS Clinical Trials Group instrument. Subjects reported the names of their antiretroviral medications as well as the number of doses and the total number of pills prescribed and taken daily [21]. The 3-day self-reported number of pills missed was computed for each HIV medication. Adherence was defined as a proportion of prescribed doses taken (0–1).

### Other factors

Basic demographic information included each participant's age in years, race/ethnicity (4 groups: black, white, Latino, other), and gender. Homelessness was defined as spending at least one night in a shelter or on the street in the 6 months prior to the interview. The number of doses of therapy each subject received per day was also recorded. A group of 151 subjects in the cohort participated in a randomized controlled trial of a HAART adherence intervention [22]. Involvement in the ADHERE trial was included as a three-category variable in our analysis (intervention/control/not enrolled). Another three-category variable was included that described a subject's primary HIV risk factor: injection drug use, men having sex with men, or heterosexual sex. Finally, to assess whether there were any important cohort effects due to date of entry in the study, we categorized subjects according to the year in which they entered the study (1997–2001).

In this secondary analysis we wanted to replicate the results of Samet et al [5] relating to adherence and alcohol consumption, while utilizing the additional information regarding follow-up observations. In addition, we extended the previous analysis to assess whether subjects' adherence to medications was acting as an effect modifier for the association of alcohol consumption with HIV progression, i.e. whether the effect that alcohol had on RNA levels differed across degrees of HAART adherence.

### Statistical methods using linear mixed effects regression models

**Figure 1:**
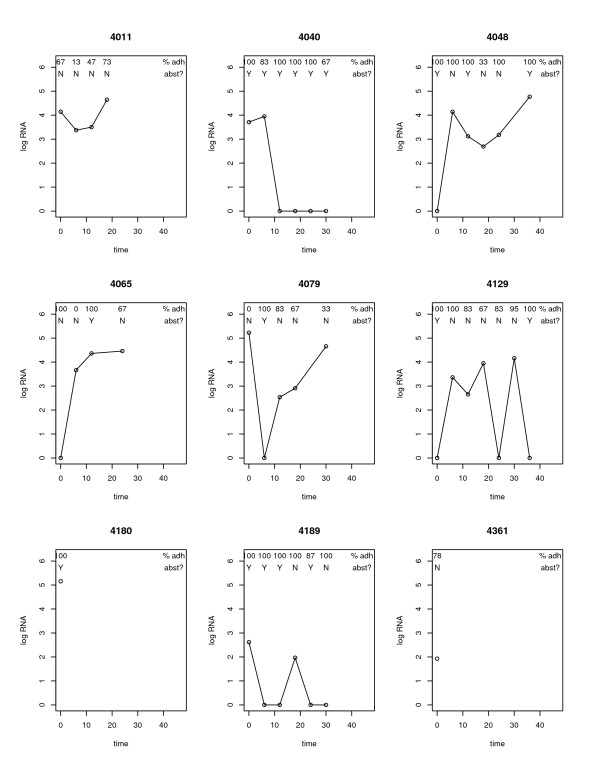
Observed log(RNA+1), adherence and abstinence status over time for 9 subjects.

We fit linear mixed effects (LME) regression models ([9,11]) for log(RNA+1) levels over time. To help ground the discussion of these models in the context of our example, we display (in Figure 1) the observed log(RNA+1) levels, adherence percentages, and alcohol abstinence values (yes/no) for a sample of nine subjects. LME models account for clustering of longitudinal data points (for example, note that subject 4011's log(RNA+1) values are consistently higher than subject 4189's) and thus provide valid estimates of the regression parameters of interest and their standard error. LME methods are also attractive in this setting because, unlike classical repeated measures ANOVA [22], they can loosen assumptions regarding the form of associations within subjects and incorporate imbalance in longitudinal data (note that subject 4180 is only observed at baseline, whereas subject 4129 has six follow-up visits). Furthermore, LME models distinguish within-subject from between-subject sources of variation, and also describe how individual and population mean response trajectories change over time. At the same time, LME models are particularly useful because their covariance structures can often be described in a flexible and parsimonious fashion.

The underlying premise of LME methods is that an outcome of interest is determined by some factors that affect all subjects in the same way and by other factors that affect individuals in different ways. This premise is reflected in the LME model by dividing the mean model regression parameters into two distinct groups: fixed effects (or population effects) and random effects (or subject-specific effects). The fixed effect parameters are shared by the entire study population. The other parameters, the random effects, are allowed to vary randomly from one individual to another. These random effects are attractive because one participant's RNA levels might consistently be higher than the mean while another's might be lower due to unmeasured factors such as genetic make-up, immunologic factors, HIV mutations conferring resistance, environment, education, personal habits, etc. These differences would be reflected in the random-effects portion of the model, thus allowing each individual to have his/her own subject-specific mean response trajectory over time. These random effects parameters reflect the natural heterogeneity of the population and thus account for within-individual clustering of data points.

In the LME framework, each participant's outcome trajectory is modeled as a combination of the population characteristics that are assumed to be shared by all individuals (fixed effects), and that individual's unique subject specific effects (random effects). The mean response trajectory in the population is obtained using a weighted average of the random effects, which are almost always a subset of the fixed effects. We will now introduce two important special cases of the LME model using the notation of Fitzmaurice et al. [9].

#### Random intercept model

The most straightforward case of a linear mixed-effects model is one in which each subject has only one random effect – a randomly determined intercept (or individual level). This model assumes that controlling for a subject's level (intercept) sufficiently accounts for the association between repeated measurements.

To illustrate this approach, consider a study with only two time-points and two levels of drinking. In this study, subject *i*'s predicted log(RNA+1) level at time-point *j *would be given by:

*E *[*RNA*_*ij*_|*t*_*i*_, *drk*_*ij*_, *b*_*i*_] = *β*_0 _+ *β*_1_*t*_*i*1 _+ *β*_2_*t*_*i*2 _+ *β*_3_*drk*_*ij *_+ *b*_*i*_

◦ *β*_0 _is the population's average intercept.

◦ *t*_1 _and *t*_2 _are dummy variables for time; *β*_1 _and *β*_2 _are their associated fixed effect regression parameters.

◦ *drk*_*ij *_= 1 if person *i *was using alcohol at time *j*, *drk*_*ij *_= 0 if person *i *was abstaining at time *j*; *β*_3 _describes the population effect of alcohol use on log(RNA+1) levels.

◦ *b*_*i *_is person *i*'s random intercept. In particular, *b*_*i *_represents the deviation of the *i*^*th *^individual's intercept from the population's intercept *β*_0_.

By averaging over the distribution of the subject-specific effects *b*_*i*_, we obtain the mean response profile in the population characterized by the fixed effect regression parameters of interest:

RNAj¯=β0+β1ti1+β2ti2+β3drkij.
 MathType@MTEF@5@5@+=feaafiart1ev1aaatCvAUfKttLearuWrP9MDH5MBPbIqV92AaeXatLxBI9gBaebbnrfifHhDYfgasaacH8akY=wiFfYdH8Gipec8Eeeu0xXdbba9frFj0=OqFfea0dXdd9vqai=hGuQ8kuc9pgc9s8qqaq=dirpe0xb9q8qiLsFr0=vr0=vr0dc8meaabaqaciaacaGaaeqabaqabeGadaaakeaadaqdaaqaaiabdkfasjabd6eaojabdgeabnaaBaaaleaacqWGQbGAaeqaaaaakiabg2da9GGaciab=j7aInaaBaaaleaacqaIWaamaeqaaOGaey4kaSIae8NSdi2aaSbaaSqaaiabigdaXaqabaGccqWG0baDdaWgaaWcbaGaemyAaKMaeGymaedabeaakiabgUcaRiab=j7aInaaBaaaleaacqaIYaGmaeqaaOGaemiDaq3aaSbaaSqaaiabdMgaPjabikdaYaqabaGccqGHRaWkcqWFYoGydaWgaaWcbaGaeG4mamdabeaakiabdsgaKjabdkhaYjabdUgaRnaaBaaaleaacqWGPbqAcqWGQbGAaeqaaOGaeiOla4caaa@5046@

**Figure 2:**
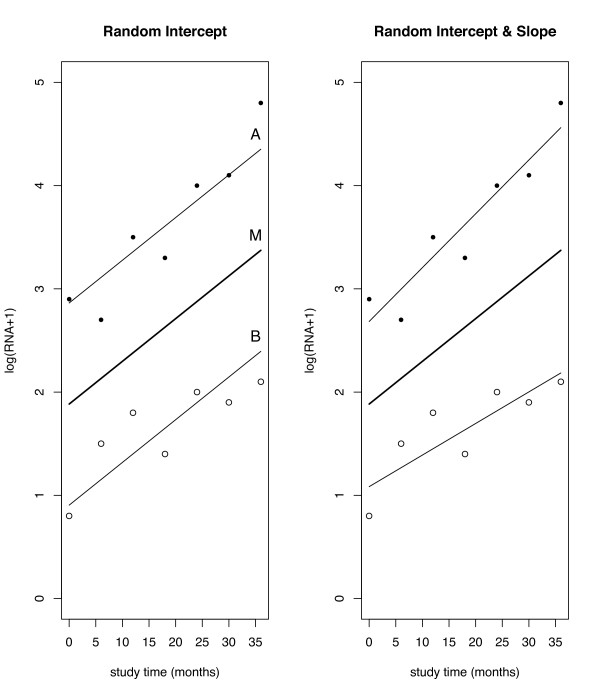
Hypothetical observed and predicted lines for two subjects from random intercept and random slope model.

In Figure 2, we use an illustration due to Fitzmaurice et al. [9] to demonstrate how this model could be applied in a simple example. In this example, as shown on the left side of the Figure, person A's measurements are consistently higher than person B. Thus, person A would have a positive random intercept term (*b*_*A *_> 0) whereas person B's random intercept would be negative (*b*_*B *_< 0). The individuals' observed responses are allowed to vary randomly above and below their conditional mean trajectories because of the inclusion of the error terms *e*_*ij*_. (In this example, *e*_*A*1 _is positive whereas *e*_*A*2 _is negative.) By averaging over these random effects, we obtain the marginal mean response trajectory, M, the predicted outcome trajectory for an 'average' subject in the population, described using the fixed effects parameters.

The usefulness of the random intercept model however, is limited by the fact that this model constrains the correlation between repeated measurements to be the same no matter how close or far apart in time the measurements are taken. For example, in a study with six time-points, the random intercept model makes the restrictive assumption that the correlation between subject *i*'s first and second measurements is the same as the correlation between subject *i*'s first and sixth measurements: *corr *(*Y*_*i*1_, *Y*_*i*2_) = *corr *(*Y*_*i*1_, *Y*_*i*6_). This assumption of time-invariant correlation is likely to be unrealistic for repeated observation of HIV RNA (e.g. we would expect two consecutive measurements to be more tightly correlated than two measurements taken far apart in time). We will now introduce a more flexible model that allows correlations between repeated measures to change over time.

#### Random intercept and slope model

Another set of random effects covariance structures, which makes less restrictive assumptions about the associations between measurements, arises when additional parameters (besides the intercept) are considered random and subject-specific. The random intercept and slope model allows both the intercept and slope to vary randomly among subjects. The model given above would thus change slightly:

*E *[*RNA*_*ij*_|*t*_*i*_, *drk*_*ij*_, *b*_*i*_] = *β*_0 _+ *β*_1_*t*_*i*1 _+ *β*_2_t_*i*2_+ *β*_3_*drk*_*ij *_+ *b*_0*i *_+ *b*_1*i*_*t*_*ij*_

◦ *b*_0*i *_is subject *i*'s random intercept.

◦ *b*_1*i *_is subject *i*'s random slope.

The random intercept and slope model can best be understood by considering another simple two-person experiment, depicted on the right side of Figure 2, again due to Fitzmaurice et al. [9]. In this example, person A's intercept and slope are greater than the mean intercept and slope respectively (*b*_0*A *_> 0, *b*_1*A *_> 0) because his or her measurements are on average higher than the average in the group and are also increasing at a faster rate. By the same token, the intercept and slope of person B are less than the population averages for intercept and slope (*b*_0*B *_< 0, *b*_1*B *_< 0). So, the mean trajectory has intercept *β*_0 _and slope *β*_1_; person A has intercept *β*_0 _+ *b*_0*A *_> *β*_0 _and slope *β*_1 _+ *b*_1*A *_> *β*_1_; person B has intercept *β*_0 _+ *b*_0*B *_<*β *_0 _and slope *β*_1 _+ *b*_1*B *_<*β*_1_. As was the case in the random intercept model, the inclusion of the error terms *e*_*ij *_allows the observed measurements to deviate randomly from the subject-specific trajectories.

Importantly, the covariance structure of this model is less restrictive than the random intercept model. In particular, the random intercept and slope model allows correlations between measurements to change with time – the correlation between *Y*_*ij *_and *Y*_*ik *_is modeled as a function of the times of measurement. To illustrate the increase in flexibility that comes from including a random slope, we return to the example of the HIV-ALC dataset, noting that in the random-intercept model, the correlation between any two measurements on the same person, regardless of how far apart they were in time, was constrained to be *corr *(*Y*_*ij*_, *Y*_*ik*_) = 0.40. The addition of a random slope, however, allowed this quantity to vary according to the times of measurement: in the random-intercept-and-slope model, *corr *(*Y*_*i*0_, *Y*_*i*6_) = 0.47 and *corr *(*Y*_*i*0_, *Y*_*i*36_) = 0.16. This agrees with intuition – one would expect measurements of viral RNA made 6 months apart in time to be more tightly correlated than measurements made three years apart. In addressing the HIV data analysis with repeated measurements taken months apart, this is an attractive feature, and we adopted this approach.

Estimation of the regression parameters of interest as well as the variance-covariance matrix of the random effects (assuming multivariate normality) proceeds simultaneously. Two options for maximization include a standard likelihood or a restricted likelihood (REML), where the former is biased in small samples. An extensive discussion of estimation can be found in [11].

It is often the case that primary scientific interest lies in the interpretation of the parameters that describe the mean and subject-specific trajectories. An appealing feature of LME models is that after the within-subject association has been accounted for (using either the random intercept model, the random intercept and slope model, or a more complex model), the nuisance parameters that describe this covariance structure can typically be ignored and focus can be given to interpreting the substantive parameters. Settings where the variance parameters are of interest in their own right (such as studies of observer variation) can be accommodated by LME models as well.

While we have focused on models for responses that are approximately Gaussian, extensions to other types of outcomes (e.g. counts or dichotomous variables) have been undertaken using the mixed effects framework. The text by Fitzmaurice, Laird and Ware [9] provides an accessible introduction to random effects for the generalized linear model.

We now turn to the specification of the log(RNA+1) regression model for the fixed effects parameters. The inclusion of follow-up data in our analysis enabled us to study the effect of time on HIV RNA levels as well as the interaction between time and alcohol use, our covariate of primary interest. Entry into the study was not linked to any treatment or clinical event, and we therefore had no specific hypotheses about relationships among time, time-varying measures of alcohol consumption and adherence, and changes in log(RNA+1) viral loads.

We included a relatively rich set of covariates into our linear mixed effects model, incorporating: time (df = 6), age, race/ethnicity (df = 3), gender, any report of homelessness, number of doses of HAART prescribed per day, adherence to HAART, involvement in the ADHERE study (df = 2) [22], year of entry into the HIV-ALC study, and primary HIV risk factor. We justified the use of this 'inclusive approach' [23] in two ways. First, any of these variables could potentially be a confounder of the relationship of interest. Second, in a study in which only 44% of intended observations were made, the inclusion of what Collins et al. [23] call 'auxiliary variables' is suggested. While the focus of this paper is not on missing data, we note that LME methodology yields consistent estimates when missing data are *missing at random *in the sense of Little and Rubin [24]. Informally, this means that missingness is ignorable when it is related only to observed quantities. By incorporation of additional information already collected in the study, assumptions regarding the ignorability of missing data become more plausible (yet we note that the validity of the assumption of ignorability remains inherently untestable without additional data regarding missing observations).

Pairwise interactions between time, alcohol consumption and adherence were included in the model, and retained if their p-values were less than 0.10. These interactions were considered because there was substantive interest in these factors. Because of the complications in interpreting multiple degree of freedom interactions, only those achieving a modest degree of statistical significance were included in the final model.

In the cross-sectional analysis conducted by Samet et al. [5], controlling for adherence to HAART yielded attenuated results (p = 0.04 as opposed to p = .006). To further explore whether adherence might be an effect modifier of the drinking/RNA association (i.e. whether the effect of drinking on RNA was modified depending on the values of adherence), we decided to test for the significance of the interaction effect between alcohol use and adherence in our model. This effect was moderately significant (p = .02) and the interaction was retained. R version 2.4.1 and SAS version 9.1 were used for estimation. The Appendix provides the syntax needed to fit the LME model in three general purpose statistical packages: R, SAS and Stata.

## Results

Table 1 describes the analytic sample of the HIV-ALC cohort on HAART. Key characteristics include the following: 58% report injection drug use as their primary HIV risk factor; 22% are homeless; 40% currently using alcohol, 18% are female, and the average age is 40 years.

**Table 1:** Characteristics of the HIV-ALC Cohort on HAART at baseline (n = 197)

	Percent	Count
Primary HIV risk factor		
Men sex with men	21%	42
Injection drug use	58%	115
Heterosexual sex	20%	40
Race/ethnicity		
Black	41%	80
White	37%	73
Latino	22%	43
Other	1%	1
Uses alcohol	40%	79
Female	18%	36
Homeless	22%	43
Enrollment year		
1997	10%	19
1998	33%	65
1999	37%	72
2000	16%	32
2001	5%	9
ADHERE enrollment		
Not enrolled	49%	96
Control	26%	52
Intervention	25%	49
	Mean (SD)	min, max
Doses of HAART/day	5.0 (1.6)	2, 10
3 day HAART adherence	0.9 (0.2)	0, 1
Age	40.8 (7.4)	19.5,66.2
Log_10_(RNA+1)	2.0 (1.9)	0, 5.7

Note: due to rounding some values may not sum to 100%

**Figure 3:**
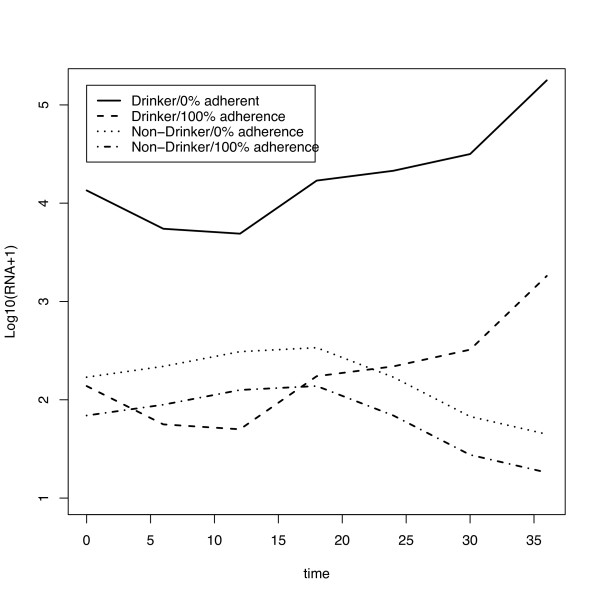
log(RNA+1) predicted values over time from random intercept and slope model by adherence and abstinence status.

Table 2 displays the results of the multiple longitudinal LME regression model of log(RNA+1) levels. Alcohol use was a significant predictor of log(RNA+1) levels with abstainers having lower levels of RNA on average. There was a significant interaction between alcohol consumption and adherence (p = 0.02) and the interaction was retained. Subjects who used alcohol and were less adherent to their medications had significantly higher log(RNA+1) levels than non-drinkers who had better adherence to their HAART regimens, alcohol users who had better adherence to their HAART regimens, and non-drinkers who were less adherent to their HAART regimens. To help interpret the statistically significant interaction between alcohol consumption and adherence, Figure 3 shows the predicted log(1 + RNA) trajectories over time for four hypothetical subjects, one who doesn't drink and is 100% adherent, one who doesn't drink and is 0% adherent, one who uses alcohol and is 100% adherent, and one who uses alcohol and is 0% adherent (each hypothetical subject represents an 'average' subject with respect to baseline covariates). The time by adherence interaction effect was dropped as there was little evidence that it added to the model (p = 0.65).

**Table 2:** Summary of LME Model of Log_10_(RNA+1) (n = 618 observations derived from 197 subjects)

	Est (SE)	p-value	Multiple *df *p-value
Intercept	1.9 (.98)	.06	
Time			.37 (*df *= 6)
Time0	.58 (.48)	.23	
Time6	.69 (.48)	.15	
Time12	.84 (.48)	.08	
Time18	.88 (.50)	.08	
Time24	.58 (.49)	.24	
Time30	.18 (.49)	.72	
Time36	0	.	
Drink	3.6 (.97)	.0003	
Adherence	-.39 (.58)	.50	
Time*Drink			.01 (*df *= 6)
Time0*Drink	-1.7 (.81)	.04	
Time6*Drink	-2.2 (.81)	.006	
Time12*Drink	-2.4 (.82)	.003	
Time18*Drink	-1.9 (.82)	.02	
Time24*Drink	-1.5 (.84)	.07	
Time30*Drink	-.93 (.85)	.27	
Time36*Drink	0	.	
Drink*Adherence	-1.6 (.67)	.02	
Age	-.02 (.01)	.12	
Female	.21 (.27)	.45	
Homeless	.04 (.19)	.85	
Doses/day	-.02 (.05)	.65	
Enrollment year	.22 (.11)	.04	
Race/ethnicity			.22 (*df *= 3)
Black	.21 (.23)	.37	
Latino	.04 (.28)	.88	
Other	1.8 (.93)	.05	
White	0	.	
ADHERE assignment			.27 (*df *= 2)
Non ADHERE	-.39 (.24)	.11	
ADHERE treatment	-.16 (.25)	.17	
ADHERE control	0	.	
Primary HIV risk factor			.25 (*df *= 2)
Men sex with men	.46 (.33)	.17	
Injection drug use	.44 (.27)	.11	
Heterosexual sex	0	.	

To assess the value of incorporating follow-up data, we also fit a cross-sectional model utilizing only data from the baseline timepoint (n = 197, Table 3). The significance of the alcohol consumption by adherence interaction was attenuated (p = 0.053), in part due to the reduced sample size.

**Table 3:** Summary of Linear Regression Model of Log_10_(RNA+1) at baseline (n = 197 observations)

	Est (SE)	p-value	Multiple *df *p-value
Intercept	1.0 (1.3)	.43	
Drink	2.6 (1.2)	.03	
Adherence	-.37 (1.0)	.71	
Drink*Adherence	-2.4 (1.2)	.05	
Age	0.0 (.02)	.98	
Female	.58 (.37)	.12	
Homeless	.44 (.32)	.18	
Doses/day	-.07 (.09)	.43	
Enrollment year	.32 (.14)	.02	
Race/ethnicity			.94 (*df *= 3)
Black	.06 (.32)	.84	
Latino	.06 (.36)	.88	
Other	1.2 (1.8)	.53	
White	0	.	
ADHERE assignment			.36 (*df *= 2)
Non ADHERE	-.12 (.33)	.73	
ADHERE treatment	.35 (.37)	.35	
ADHERE control	0	.	
Primary HIV risk factor			.13 (*df *= 2)
Men sex with men	.83 (.7)	.08	
Injection drug use	.69 (.37)	.06	
Heterosexual sex	0	.	

We also assessed the importance of accounting for clustering, by fitting a model for all time points that inappropriately ignored the correlation. This incorrect model yielded a spuriously statistically significant interaction effect for alcohol consumption and adherence (p = 0.0006).

**Figure 4:**
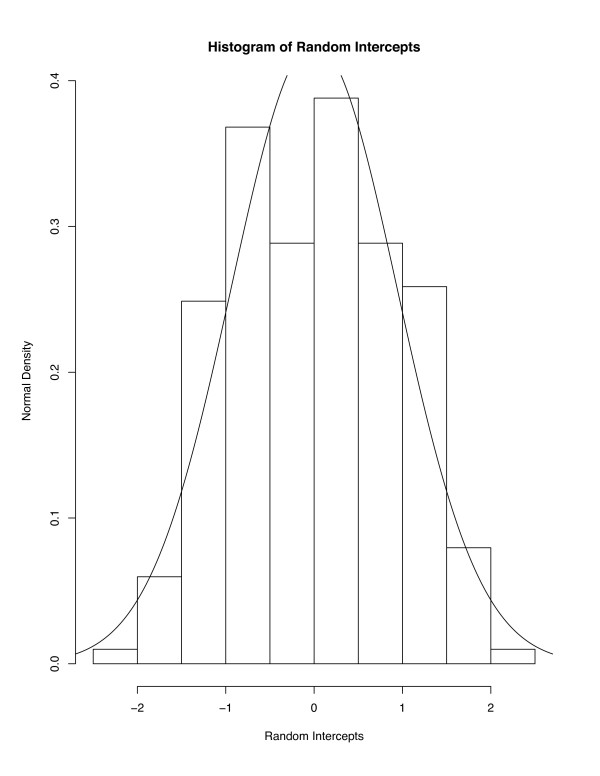
Histogram of random intercepts from random slope model (plus normal density).

**Figure 5:**
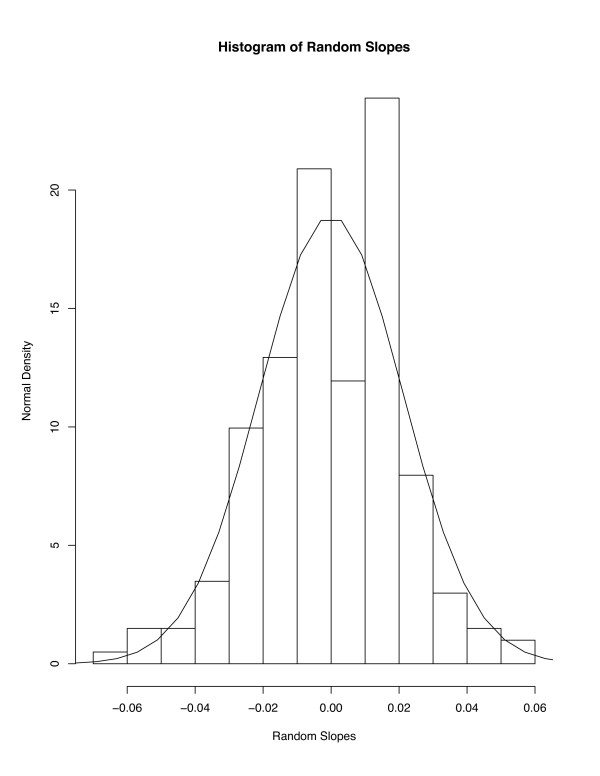
Histogram of random slopes from random slope model (plus normal density).

For any model, it is important to verify assumptions made in estimation. For the LME random intercept and slope model, in addition to assumptions of standard multiple regression models, estimation proceeds assuming that the distribution of the random intercepts and slopes is approximately bivariate normal. Figure 4 displays a histogram (with normal [mean = 0, variance = 1.5] density overlaid) of the random intercepts, while Figure 5 displays the histogram of the random slope parameters (with normal [0,0.0019] overlaid). Neither histogram presents strong evidence against the normality assumption.

**Figure 6:**
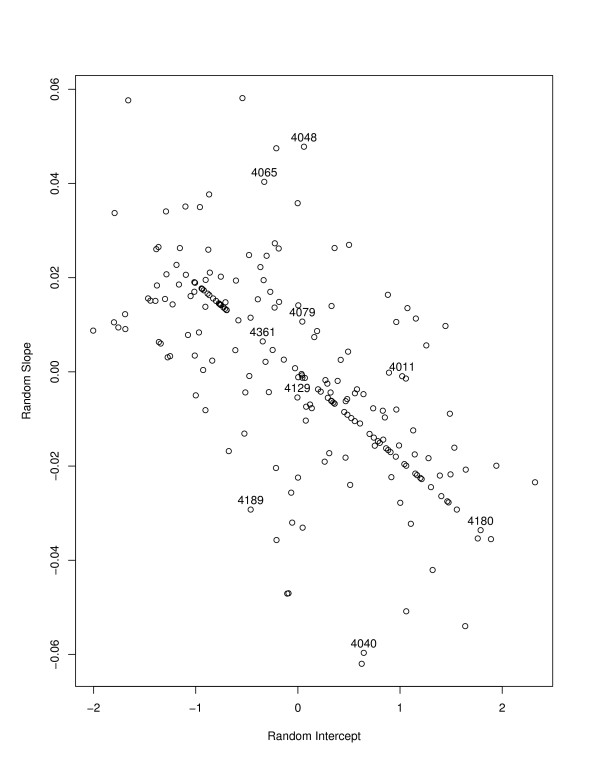
Scatterplot of random intercepts and random slopes (9 subjects displayed in Figure 1 are indicated by their identification numbers).

Figure 6 displays the scatterplot of random slopes and intercepts, which appears to be a cloud of points consistent with a bivariate normal density, albeit with a number of points on the line given by slope = -.02*intercept. The 9 subjects labeled in Figure 1 are indicated to help interpret this scatterplot. For subjects with only one observation (e.g. 4180, 4361) the predicted slope is essentially *borrowed *from other values within the sample. Many of the subjects in the HIV-ALC cohort were observed at only 1 timepoint. The negative correlation (-0.53) of the cloud of points indicates that there is an inverse association between intercepts and slopes: subjects with low log(RNA+1) values at baseline are likely to see increases in log(RNA+1) values over time (i.e. have more potential for increase), while subjects with high log(RNA+1) values at baseline are likely to decrease over time. Subjects whose log(RNA+1) values increased over time had positive random slopes (e.g. 4065, 4048) while subjects with a decrease had negative slopes (e.g. 4189 and 4140).

## Conclusion

Our primary goal was to motivate and illustrate the use of linear mixed effect regression models for longitudinal epidemiologic data in an alcohol research setting. Using the LME model to account for clustering within subject enabled us to make full use of the available data. Whereas Samet et al. [5] conducted a cross-sectional analysis of the baseline data from the HIV-ALC cohort, we analyzed data collected at 7 different timepoints. By utilizing this additional information, we were able to detect the subtle interaction effect between adherence and alcohol consumption that was moderately significant in our longitudinal analysis (p = .018), but only borderline significant (p = .053) in a cross-sectional baseline analysis that included all other covariates except time. While the substantive conclusions regarding this interaction are similar in both models, the potential efficiency gain of including all available observations should not be dismissed. The results of our analysis suggest that for this sample of subjects with a history of alcohol problems either adhering to one's HAART regimen, or abstaining from alcohol was significantly associated with relatively lower levels of log(RNA+1) viral load. Limitations of this investigation include self-report of alcohol consumption and adherence, missing data, and relatively modest sample size. We also note the possibility that effects of readiness to change as a predictor of subsequent drinking behavior may be mediated/moderated by factors such as "self-efficacy," which the current analyses do not directly address. Further exploration of the interacting effects of drinking and adherence thus seems merited.

The linear mixed effects models that we have described provide a flexible structure for modeling the covariance among repeated observations, thus yielding valid estimates of regression parameter variances. We concur with the advice of Fitzmaurice and colleagues who stated that given modern computing capabilities, which support a wider class of models for longitudinal data, "there is little reason to analyze longitudinal data under the inherent limitations and constraints imposed by the repeated measures ANOVA model" [9].

After the association in longitudinal studies has been accounted for, focus can shift to interpreting the substantive parameters that describe the relationships of scientific interest. The interpretation of parameters from a multiple regression model is of crucial importance, and a similar process of interpretation is needed for the LME model.

Extensions to non-normally distributed outcomes (e.g. binary or count outcomes), while not discussed in detail here, are tractable, as routines to fit both linear and non-linear models exist in general purpose statistical software (including but not limited to R, SAS, S-plus, SPSS, and Stata). While there are a number of additional complications in fitting non-linear models, in terms of computational requirements and convergence, the general framework is analogous to the linear setting that we describe. These models are applicable to a wide range of outcomes arising in alcohol studies, and should be utilized routinely.

Incomplete observations arise in most longitudinal studies. It is rarely, if ever, the case that every planned measurement can be successfully obtained; some subset of these intended measurements are often missing. LME methods incorporate incomplete data under the assumption that missingness is at random (MAR, not related to unobserved quantities). Although estimates made under MAR have been shown to be relatively robust to small deviations from this assumption [23] this is not always true, and it is important to consider whether this (untestable) assumption is tenable. An extensive literature exists regarding the use of non-ignorable non-response models to assess sensitivity to the MAR assumption [25].

As with any model, verification of other assumptions (residual analysis, examination of influential points, etc.) is critically important. We focused attention on assumptions of normality of the random effects parameters, but other model-checking is always indicated. The multiple-bias methods of Greenland [26] provide a general framework for consideration of non-sampling errors that could affect results in substantial ways.

In this report we have provided a brief introduction and application of LME models, but have only touched on many important issues and have neglected other crucial aspects. More comprehensive descriptions of these methods exist (e.g. [8,9]) and are appropriate next steps for analysts considering use of these models. In addition, other approaches to the analysis of longitudinal or clustered data have been proposed. The population averaged generalized estimating equation (GEE) approach of Liang and Zeger [27] is another feasible approach, particularly for non-normally distributed outcomes. Extensions of the random effects framework using Bayesian estimation [28] have been utilized to address additional complexities and loosen assumptions (i.e. use of a t distribution rather than a normal distribution for the random effects). Finally, latent variable models [29] provide an attractive framework with a similar flavor, with implementations available (e.g. Stata and Mplus). These models extend the random effects model to fit multilevel factor and item response models, latent class models, and multilevel structural equation models.

## Competing interests

The author(s) declare that they have no competing interests.

## Authors' contributions

MF and NH designed and carried out the secondary analysis. MF helped to draft the manuscript. JS conceived of the original study, participated in its design and coordination. All authors contributed to the creation of earlier drafts, and all read and approved the final manuscript.

## Appendix

Code to implement linear mixed effects models in general purpose statistical software.

R

1 library(nlme)

2 lmefit < – lme(logrna ~as.factor(time) + drkhaz2 + pct3d +

3      as.factor(time)*drkhaz2 + drkhaz2*pct3d + age +

4      as.factor(race) + female + homeless + dose_day +

5      as.factor(adhere3) + as.factor(hivrsk) + cohort2,

6      data = ds, random = ~time2 | patid)

In line 1, we load the "nlme" (non-linear mixed effects) library. In line 2, we use the command "lme" to fit an LME model with response variable "logrna", with output object "lmefit". Lines 2–5 specify the model's fixed-effects covariates, with categorical variables designated using "as.factor". In line 6, we specify the analysis dataset, and indicate that subject's slopes over "time2" (a continuous version of time) are to be random. To indicate the subject clustering, we specify the subject ID number variable, as the grouping factor, using the code "| patid".

SAS

7   proc mixed data = ds;

8      class patid time hivrsk race adhere3;

9      model logrna = time drkhaz2 pct3d time*drkhaz2

10         drkhaz2*pct3d age race female homeless dose_day

11         adhere3 hivrsk cohort2/s;

12      random int time2/type = un subject = patid s g;

13 run;

In line 7, we call SAS PROC MIXED, applying it to a dataset called "ds". In line 8, we specify the categorical variables. In lines 9–11, we use the "model" statement to specify our response variable and fixed effects, with "/s requesting the regression solution. In line 12, we specify a random intercept and slope model using a continuous time variable ("time2") and that the data is clustered by the subject ID variable "patid", with an unstructured working covariance matrix for the random effects parameters.

Stata

14 xi: xtmixed logrna i.time drkhaz2 pct3d i.drkhaz2*i.time

15      i.drkhaz2*pct3d age i.race female homeless

16      dose_day i.adhere3 i.hivrsk cohort2 || patid: time2,

17      covariance(unstructured)

The "xi" command allows the dynamic creation of categorical variables as well as interactions for the "xtmixed" command. The clustering is indicated by the "|| patid:" command, with "time2" given as the continuous measure of time and unstructured working covariance matrix for the random effects parameters.
